# KEGGscape: a Cytoscape app for pathway data integration

**DOI:** 10.12688/f1000research.4524.1

**Published:** 2014-07-01

**Authors:** Kozo Nishida, Keiichiro Ono, Shigehiko Kanaya, Koichi Takahashi

**Affiliations:** 1 Laboratory for Biochemical Simulation, RIKEN Quantitative Biology Center, Osaka, 565-0874, Japan; 2 JST, National Bioscience Database Center (NBDC), Tokyo, 102-0081, Japan; 3 Department of Medicine, University of California San Diego, La Jolla, CA 92093, USA; 4 Graduate School of Information Science, Nara Institute of Science and Technology, Nara, 630-0101, Japan

## Abstract

In this paper, we present KEGGscape a pathway data integration and visualization app for Cytoscape ( http://apps.cytoscape.org/apps/keggscape). KEGG is a comprehensive public biological database that contains large collection of human curated pathways. KEGGscape utilizes the database to reproduce the corresponding hand-drawn pathway diagrams with as much detail as possible in Cytoscape. Further, it allows users to import pathway data sets to visualize biologist-friendly diagrams using the Cytoscape core visualization function (Visual Style) and the ability to perform pathway analysis with a variety of Cytoscape apps. From the analyzed data, users can create complex and interactive visualizations which cannot be done in the KEGG PATHWAY web application. Experimental data with Affymetrix E. coli chips are used as an example to demonstrate how users can integrate pathways, annotations, and experimental data sets to create complex visualizations that clarify biological systems using KEGGscape and other Cytoscape apps.

## Introduction

Kyoto Encyclopedia of Genes and Genomes (KEGG, http://www.genome.jp/kegg) ^1^ is a widely used biological database of high-level biological functions. It contains pathway data sets that have comprehensive annotations and high quality human-curated, hand-drawn diagrams. Most biological pathway databases store data as machine-readable graph topologies, which leave much of the details about how the diagrams were drawn excluded from the data files. This is a problem when third-party developers want to reproduce the pathway diagrams in their applications. In contrast, the KEGG PATHWAY database stores graphics information in machine-readable KEGG Markup Language (KGML, http://www.kegg.jp/kegg/xml) format. Thus, in these pathway diagrams, biological entities, such as enzymes or compounds, are manually laid-out and the diagrams are easy to understand for biologists.

The KEGG PATHWAY database is deployed as a web application using static bitmap images for pathway diagrams, and user-provided date is integrated with KEGG Mapper ( http://www.genome.jp/kegg/mapper.html). Furthermore, KEGG Atlas ( http://www.genome.jp/kegg/atlas.html) provides a comprehensive network view of global metabolic pathways. Recent improvements to KEGG Atlas, such as Pathway Projector ^2^ and iPath2 ^3^, have made it possible to perform basic data integration and visualization like mapping the expression values to node graphics. However, despite these features, it is difficult to integrate external data sets and create custom visualization. Furthermore, they are limited to those on existing desktop pathway analysis applications. To ameliorate these problems, several projects for integrating a user’s own models onto the KEGG pathways have therefore been developed (CytoSEED Cytoscape app ^4^, KEGGtranslator ^5^).

Cytoscape ^6, 7^ is a de-facto standard software platform for biological network analysis and visualization. One of its advantages is its large collection of apps for a variety of biological problem domains, such as Gene Ontology term enrichment analysis (BiNGO ^8^) and statistical network analysis (CentiScaPe ^9^), which are also mostly open source software. Additionally, Cytoscape has a flexible network visualization function and is optimized for large-scale network analysis. There are several applications dedicated to biological pathway analysis (Vanted ^10^, VisANT ^11^) that support KGML by default. Although Cytoscape does not have a built-in function to load biological pathways, if this task is done with a separate app, users can take advantage of its large-scale network analysis features, variety of analysis apps, and data visualization of biologists-friendly human curated pathways.

**Figure 1.**
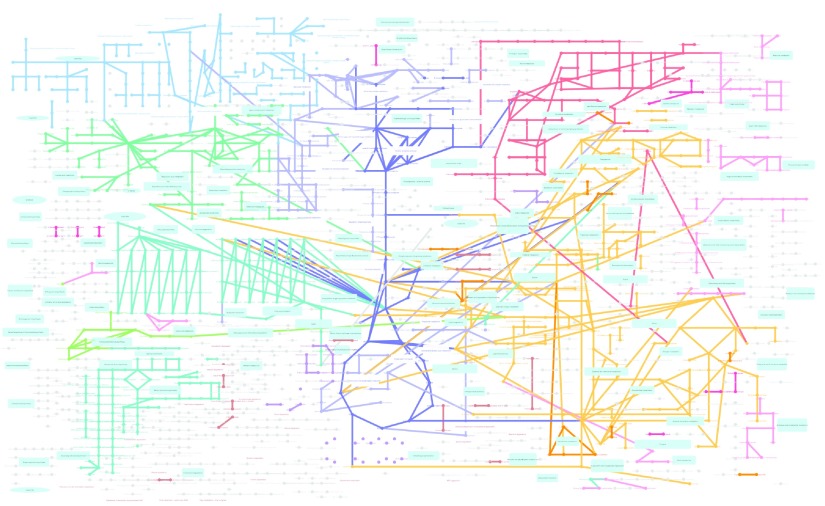
A KEGG Global Metabolic Pathway generated with the KEGGscape app.

The goal of our new Cytoscape app, KEGGscape, is to bridge the flexibility of fully-featured network analysis platforms with the high-quality pathway diagrams available in the KEGG PATHWAY web application. KEGGscape, a successor of KGMLReader ( http://apps.cytoscape.org/apps/kgmlreader) for Cytoscape 2 series, is an app that imports KEGG pathway diagrams from KGML files and provides a new way to use KEGG pathway diagrams as data integration blueprints in cooperation with Cytoscape core features and an existing variety of apps. KEGGscape is completely re-designed for the new Cytoscape 3 API and supports signaling pathways in addition to metabolic pathways, including the global metabolic pathways used in KEGG Atlas ( Figure 1). In this paper, we present a basic design and implementation of KEGGscape and an example workflow utilizing KGML files, and experimental data to create information-rich pathway visualizations for clarifying omics-scale data sets. KGMLReader is the first open-source Cytoscape app that reads the graphics details of KGML files, and KEGGscape was designed to use standard Cytoscape features only. These feature enable users to use KEGG pathways with other data sets easily.

## Implementation

KEGGscape is a Cytoscape 3 app written in Java programming language and is designed to load pathway data files in KGML format. KGML is an XML file format designed by the KEGG project and contains the topology of pathways and visual representations of all elements in the diagram. KGML has formal specification as a DTD (Document Type Definition) file, which enables the use of unmarshaller ( https://jaxb.java.net) for converting XML elements directly into Java objects. This conversion creates two types of data: pathway topology and its graphical representations. Pathway topology and its properties are converted into CyNetwork and CyTable objects, which are the standard data model in Cytoscape 3. In KGML, all graphical information, such as the color of enzymes or shape of compounds is stored under <graphics> tag. Instead of setting the graphics details of nodes and edges directly from this information, Cytoscape generates Visual Style, which is a collection of default visual properties and visual mapping function, for each pathway based on the information under this tag. KEGGscape follows a standard CyNetworkReader design guideline, which enables Cytoscape to detect KGML files automatically.

### Workflow

**Figure 2.**
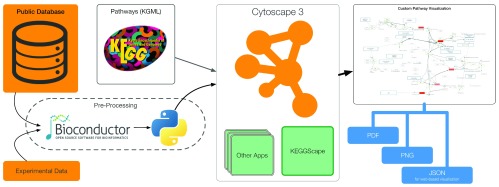
Basic pathway analysis workflow with KEGGscape. Cytoscape with KEGGscape can be used as a part of larger workflows to publish integrated pathway visualizations as vector graphics, bitmap images, or JSON for web-based visualization using Cytoscape.js ( http://cytoscape.github.io/cytoscape.js/).

Figure 2 shows an example of a pathway analysis workflow with KEGGscape. To take advantage of the flexible visualization and analysis features in Cytoscape, users need to import as much information as possible for the pathways they want to analyze. Although Cytoscape is a powerful tool for biological data integration, it is not the best platform for data preparation or cleansing. Users can instead prepare annotations and experimental data sets for the pathway using tools of their choice, such as R (Bioconductor ^12^), Python, or Excel. Once the data files are ready, Cytoscape can read them into an on-memory session and visualize the data on the KEGG pathways. Imported data sets only use standard Cytoscape data objects, and users can then access all of the standard Cytoscape features to create custom pathway visualizations. An actual workflow will be presented in a later section.

### Limitations

**Figure 3.**
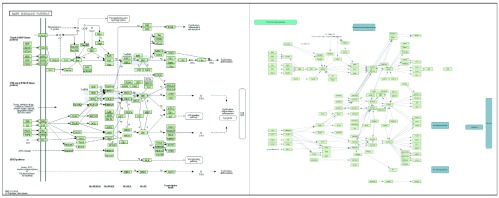
Comparison of the original diagram and Cytoscape visualization for the human MAPK signaling pathway (KEGG hsa04010).

Although KEGGscape can read all information of the pathways saved in KGML files, some of the pathway visualizations in Cytoscape look slightly different from the original hand-drawn diagrams available on the KEGG website. The cause of this issue is missing graphics information in the KGML files. Figure 3 is a side-by-side comparison of the same pathway visualization (human MAPK signaling pathway; KEGG ID: hsa04010). The original diagram (left) contains several background visual annotations that are not visible in the visualization created by Cytoscape (right). The hand-drawn compartmental annotations are not encoded in KGML files, which means they cannot be reproduced by KEGGscape.

## Results

As an example workflow, we integrated and visualized a KEGG pathway and gene expression profile using KEGGscape and external tools. In this example, the differentially expressed genes between two groups, mutants and controls, in a global expression profile are mapped on the KEGG pathway, as too are the t-test results.

### Data preparation

To perform this pathway analysis in Cytoscape, we used Bioconductor ( http://www.bioconductor.org/) to prepare the gene expression matrix data. We normalized Affymetrix GeneChip data by the robust multi-array average (RMA) method with the Bioconductor packages ecoliLeucine ^13^ and affy ^14^. The leucine regulatory protein (Lrp) is a DNA binding protein and known as a leucine responsive global regulator ^15^. The p-value for each probeset between four lrp mutant strains and four control chips was calculated by rowttest method in genefilter package ^16^. From this calculation, we obtained a list of genes that are differentially expressed (p-value < 0.05). We sent these probeset identifiers to KEGG Mapper and picked the highest hit, which was the glycine, serine and thereonine metabolic pathway (KEGG ID: eco00260) for visualization.

### Visualization

**Figure 4.**
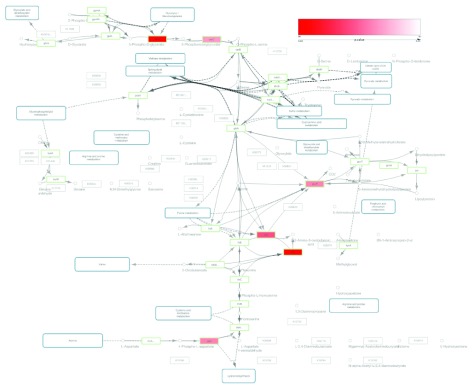
KEGG pathway visualization integrated with gene expression data for the glycine, serine and threonine metabolism pathway of Escherichia coli K-12 MG1655 (KEGG eco00260). Green border nodes are KO (KEGG Orthology) annotated nodes. Red colored nodes include differentially expressed genes (p-value < 0.05).

To create a visualization using all the data sets, we imported the KGML file of eco00260 and the p-value matrix file prepared in the previous data preparation to Cytoscape 3, and merged the matrices with a custom Python script ( Figure 4). Because Cytoscape does not support fuzzy key matching, we used our Python script to append a key column to the p-value matrix to utilize the Cytoscape table merge tool.

The node table in Cytoscape for the imported KGML had KEGG gene annotations. The gene IDs for each enzyme node were used as keys for merging the KGML node table and p-value matrix. In this Figure 4, node colors in the original KEGG pathway were mapped to node border colors and p-values were mapped to node color gradient (red to white) to visualize the significantly expressed genes.

## Conclusions

In this paper, we presented the design and implementation of KEGGscape and an example analysis workflow integrating global gene expression profiles and KEGG pathways using KEGGscape and two external tools, Bioconductor and Python. The workflow demonstrates how users can integrate omics data in an interactive pathway diagram.

### Future plan

Current workflow can map arbitrary omics data onto interactive KEGG pathway diagrams, but it requires some manual editing to create informative visualizations. To minimize the manual process in the workflow, we plan to implement a collection of utility Python scripts to manipulate networks and Visual Styles via RESTful API, which will be published as a part of the Cytoscape 3.2.0 release. This set of Python scripts works to merge pathway related table metadata (omics profiles, non-KEGG pathway metadata) from external platforms like R and Cytoscape to automate common tasks in the visualization process.

## Software availability

The app website: http://apps.cytoscape.org/apps/keggscape

Latest source code: https://github.com/idekerlab/KEGGscape

Source code as at the time of publication: https://github.com/F1000Research/KEGGscape/releases/tag/V1.0

Archived source code as at the time of publication: http://dx.doi.org/10.5281/zenodo.10560 ^17^

License: Apache License Version 2.0
