# Supplementary material for: KEGGscape: a Cytoscape app for pathway data integration
Source: F1000Res. 2014 Jul 1;3:144. [Version 1] doi: 10.12688/f1000research.4524.1 (PMC4141640; doi:10.12688/f1000research.4524.1)
Supplement: Peer review history

# Peer review: Referee response for version 1

**DOI:** 10.5256/f1000research.4838.r5318

**Published:** 2014-08-06

**Authors:** Melissa Cline

**Affiliations:** 1 Center for Biomolecular Science & Engineering, University of California, Santa Cruz, Santa Cruz, CA, USA

KEGG is one of the foremost sources of metabolic pathway data. Cytoscape is the *de facto* standard pathway visualization platform. Cytoscape-based visualization of KEGG pathways has always been cumbersome and limited. KEGGscape gets over many of these limitations by streamlining the process, and improving the translation of the graphical elements of KEGG pathways. This makes KEGGscape a very useful resource for the scientific community.

The manuscript is clear and informative. There are only two changes I would ask for:

- First, the authors should mention that KGML files are currently freely available on the KEGG website. This will be news to some readers of this paper, since KEGG has gone through various distribution and licensing models.
- Second, the authors should expand their discussion of merging gene expression data with KGML diagrams. The power of network visualization is in pathway data integration. If there are special challenges in integrating with KEGG pathways, these should be brought forth. The mention of custom python scripts and fuzzy ID matching suggests that this integration task can involve special challenges.

I have read this submission. I believe that I have an appropriate level of expertise to confirm that it is of an acceptable scientific standard.

# Peer review: Referee response for version 1

**DOI:** 10.5256/f1000research.4838.r5317

**Published:** 2014-07-22

**Authors:** Egon Willighagen

**Affiliations:** 1 Department of Bioinformatics - BiGCaT, Maastricht University, Maastricht, Netherlands

The paper describes an interesting and relevant Cytoscape plugin; it is well written (there are a few typos), but I think it can be improved in places. An important reservation is that I cannot determine at this moment how common or serious the data loss in KGML reading is (see below). That is it should be clarified whether this paper is a first report, a work in progress or a finished project..

**Title and Abstract**

I find the title appropriate, but it may be promising more than what the app actually does. As far as I can tell, the app is not a general solution, but one specific for KEGG. The abstract is short on setting the state of the domain and, like the Introduction, does not refer to existing research in fields beyond their own. In this way, the abstract is less than clear what unsolved problem was resolved.

**Introduction**

The Introduction sufficiently explains the setting of the plugin. One thing that strikes me is the lack of references to related work. For example, it mentions " *most biological pathway databases*" without citing or even naming them, which I find suboptimal as they critique those databases and to which the authors contrast their solution. Unlike the Introduction currently seems to suggest, KGML is not the only pathway format that tracks graphical information (e.g. GPML used by PathVisio). The first paragraph currently suggests it solves a problem that others have been tackling too. (P.S. under what license is the KGML specification available?).

The Introduction focuses on technical issues of the integration. It could also say a bit about identifier mapping and the role of licensing in data integration. That is, given  the enormous impact of KEGG it can be wondered why this kind of integration with KEGG has not been done yet, because people have done this for other data sets for years (both open source and proprietary). Or, if it has, it should be cited.

Minor comments:

- Check the hyphenation of KEGGtranslator (KEGG-translator versus KEG-Gtranslator).
- The comment that Cytoscape does not have a built-in function to load biological pathways probably refers to formats, not the pathways themselves.
- Since the app focuses on KGML support, some reference to apps supporting other formats (BioPax, SGML) seems appropriate.

**Figures**

The figures are too hard to read, both in print (where it is impossible) but also in the PDF. This should really be addressed.

**Implementation**

The Implementation sections can be improved: the section is short on details on the KEGGscape source code, the design of the app and the KGML reader in particular, build instructions, development model, testing (tests seem absent in the code repository).

I also note issues with the KGML readers. For example, connections clear on the KEGG website are missing and misplaced when read into Cytoscape (e.g. between glycine and sarcosine, and the link between Purine metabolism, see Fig. 4) and labels are often misleadingly placed and often unconnected (a limitation of KEGG), though accurately copied from the KGML, it seems. The first data-loss clearly indicates a problem with the reader. These things must be discussed in the Limitations subsection, in my opinion (and/or returned to in the Future Plan at the end).

Minor comments:

- on-memory -> in-memory?
- <graphics> tag -> <graphics> element (a tag does not really have something under it in XML, attributes at best; an element does).
- What is "Visual Style"?
- Under what license is the DTD available?

**Results**

The Results section seems to accurately describe what they did, but not how they did this. I think something like a methods section (or an Open Notebook) that explains how the steps are performed (in more detail) would be helpful. However, in the end I was able to figure out how to import a KGML file (which is not under Import).

Another example is reference to a custom Python script without description. In fact, thinking about this again, for an app that does data integration, I would actually expect the app to take care of data processing as much as possible. While I understand that general preparation starting from raw data is best done in other tools, this Python script does not seem to do more than format handling of some kind. Is that correct? If so, why is this not done by the app? Fortunately, this observation was made by the authors too, and they return to it in the Future plan subsection.

Minor comments:

- probeset -> probe set
- thereonine -> threonine

**Conclusion**

The Conclusion section does not sufficiently summarize the paper: it should reiterate what the problem is that is solved and what makes KEGGscape unique. Although I do not want to imply that I think this paper is not relevant (it is)!

Also the limitations should be returned to here (those already listed in the paper, and those missing), particularly in relation to the "Future plan". The authors’ focus should really be on getting the KGML reading performing well: data loss, and data corruption are serious issues. A decent testing toolkit as Maven supports, may be a good start here, but is currently missing from the source code repository.

Minor comments:

- check the hyphenation of KEGGscape (KEGG-scape versus KEG-Gscape)

I have read this submission. I believe that I have an appropriate level of expertise to confirm that it is of an acceptable scientific standard, however I have significant reservations, as outlined above.

# Peer review: Referee response for version 1

**DOI:** 10.5256/f1000research.4838.r5316

**Published:** 2014-07-08

**Authors:** Matthew DeJongh

**Affiliations:** 1 Department of Computer Science, Hope College, Holland, MI, USA

KEGGscape is valuable Cytoscape app that enables flexible visualization and manipulation of KEGG pathway maps. The capability for visualizing data sets (e.g., gene expression levels) on pathways in Cytoscape is particularly appealing. However, the example presented in the paper does not contain sufficient information to enable a reader to duplicate the process. First, the authors do not cite the origin of the gene expression dataset they use in their example. Second, as far as I can tell, there is no tutorial provided with KEGGscape. At a minimum, the authors should provide instructions for opening a KGML file and loading a dataset.

I have read this submission. I believe that I have an appropriate level of expertise to confirm that it is of an acceptable scientific standard, however I have significant reservations, as outlined above.
